# The BUDDYS System: A Unique Peer Support Strategy Among Anaesthesiology Residents During the COVID-19 Pandemic

**DOI:** 10.5152/TJAR.2021.21247

**Published:** 2022-04-01

**Authors:** Basma A. Mohamed, W. Kirk Fowler, Mrugesh Thakkar, Brenda G. Fahy

**Affiliations:** 1 Department of Anaesthesiology, University of Florida Faculty of Medicine, Gainesville, Florida, USA; 2 Resident Wellness Program, Department of Anaesthesiology, University of Florida Faculty of Medicine, Gainesville, Florida, USA

**Keywords:** Burnout, COVID-19, graduate medical education, mental health, wellbeing

## Abstract

**Objective:**

The coronavirus disease 2019 pandemic stressed healthcare organizations. Initial efforts focused on supplies with a minimal emphasis on frontline healthcare workers’ wellbeing. Anaesthesiology residents represent vulnerable frontline healthcare workers because airway procedures increase nosocomial infection risks. Peer support can promote healthcare workers’ wellbeing during crises; its application to graduate medical trainees is underrepresented in the literature. We implemented a quality improvement project to improve wellbeing among anaesthesiology residents via a peer support system called BUilding Dynamic Duos for Your Support.

**Methods:**

BUilding Dynamic Duos for Your Support consists of pairing 2 anaesthesiology residents with instructions to support each other in anticipation of a coronavirus disease 2019 case surge. A lecture presentation introduced this system to the residents and described frequent check-ins with another resident. We evaluated the initiative with a survey 2-4 weeks postimplementation.

**Results:**

BUilding Dynamic Duos for Your Support began in April 2020 and involved 88 residents. Survey respondents (n = 58) indicated that BUilding Dynamic Duos for Your Support had a positive impact on their wellbeing. BUilding Dynamic Duos for Your Support implementation had no additional costs, requiring minimal resource dedication.

**Conclusions:**

BUilding Dynamic Duos for Your Support promoted wellbeing among anaesthesiology trainees. This quality improvement project highlights the positive impact of a peer support system on anaesthesiology residents’ wellbeing with a potential broader application to graduate medical education.

## Main Points

- Peer support can promote healthcare worker wellbeing during crises, such as the COVID-19 pandemic.
- BUDDYS is a potential wellness tool for graduate medical education that benefited the majority of our residents in an anonymous survey. This system can help prevent burnout, minimize anxiety, build resilience, and contribute to work-life balance goals for anaesthesiology residents.
- BUDDYS requires no additional financial commitment, and the necessary resources should be readily available to residency training programs.

## Introduction

As the coronavirus disease 2019 (COVID-19) outbreak rapidly became a pandemic, it created unprecedented challenges for healthcare systems around the world. Initial responses to the pandemic focused on the availability of healthcare resources, including personal protective equipment, intensive care unit beds, and medical devices, with less attention on the psychological wellbeing of frontline healthcare workers (HCWs).^1^ The impact of the pandemic on social stability and human health, as well as its economic burden, has resulted in additional challenges for frontline HCWs to remain focused on patient care while maintaining their mental, emotional, and cognitive wellbeing. Hospital systems addressed these challenges with daily and weekly meetings and huddles to provide updates, often sharing clinically relevant information without establishing clear strategies to address the overall wellbeing and mental health of HCWs. 

As frontline workers, graduate medical education trainees, especially anaesthesiology residents, are vulnerable during this pandemic. Anaesthesiology residents perform aerosol-generating procedures, including endotracheal intubation. Before the pandemic, we started several wellness initiatives in our residency program to improve wellbeing among anaesthesiology residents. These initiatives focused on burnout prevention, work-life balance, and a basic understanding of self-care; however, these initiatives were not well received and we recognized that culture change was required. When the COVID-19 pandemic began, however, the implementation of these initiatives became more urgent. The US Centers for Disease Control and Prevention’s website^2^ provide resources for clinicians and patients with an abundance of information related to COVID-19. Some healthcare organizations have encouraged peer support systems as part of a larger coping strategy, including promoting resilience and avoiding burnout in HCWs during the COVID-19 pandemic.^2-9^

As a result, our resident training program adopted the idea of peer support or the BUilding Dynamic Duos for Your Support (BUDDYS) system. The purpose was to allow residents to establish the habit of checking in on their buddy on a daily basis. In BUDDYS, 2 residents partner to support each other throughout the pandemic response and in our case, during a period of cohorting. Cohorting was an imposed grouping of anaesthesia care providers, including residents, who had the potential to be exposed while providing clinical care to patients with COVID-19. It was suggested that the 2 residents typically be in different cohorts, with 1 cohort providing clinical care and the alternate cohort performing non-clinical assignments. However, as a result of the unpredictability of pairing, this suggestion was not followed to allow for a timelier implementation of the peer support system. 

We describe the implementation of a unique approach to promote psychological and emotional wellbeing and encourage self-care approaches using a peer support system for anaesthesiology residents called BUDDYS. We describe the creation of this system as the first quality improvement project related to resident wellness in our department that resulted in promising feedback and an unexpectedly high participation rate. The purpose of this article is to highlight the initiative as the beginning of a series of wellness initiatives in the form of plan-do-study-act (PDSA) cycles within our department. The first PDSA cycle of the first quality improvement initiative focused on anaesthesiology residents. Future PDSA cycles will address further initiatives regarding the peer support system and evaluate the impact on residents’ wellbeing.

## Methods

This quality improvement project represents a qualitative design study using a survey of study participants to assess the outcome of the implementation of a peer support system. The goals of qualitative research designs are to gain insight and explore the complexity inherent in a phenomenon. Due to the crisis nature of the COVID-19 pandemic and the urgent need for resident support, we chose a qualitative design for our work. The overall aim was to implement a peer support strategy to improve anaesthesiology residents’ wellbeing during the pandemic. The BUDDYS system as a peer support strategy was initially presented to the resident wellness committee for feedback. After support was obtained from the resident wellness committee, a plan for implementation was presented and approved by anaesthesiology residency leadership. The plans for implementation involved the presentation of the BUDDYS system during a required morning didactic session for the anaesthesiology residency program as one of the coping strategies to improve wellbeing during the pandemic. 

The specific aims of BUDDYS were to introduce the concept of peer support among anaesthesiology residents, highlight the importance of self-care through building a support system, and incorporate coping strategies to navigate stressful situations at work. The didactic system presented by one of the authors (BAM) emphasized to the residents that they should be comfortable sharing concerns with their buddy, and all residents were encouraged to partner with their preferred colleague. The signs of physician distress and burnout were also covered to aid in early recognition. The presentation highlighted resources available within the training program, institution, and healthcare system in case they were needed. During the morning lecture, residents were also provided with mechanisms/resources to elevate concerns about themselves or their buddy if safety concerns arose. For a communication strategy, the resident wellness committee chairs emphasized the importance of this approach using a “group text” messaging system. A line of communication was open among all residents and the assistant program director for resident wellness with questions and an explanation of how BUDDYS would work and its potential benefits. A week after the introduction of BUDDYS, the resident wellness committee followed up with all of the residents regarding the progress of pairing. With an emphasis on the pairing process, while anticipating a COVID-19 case surge, a few residents remained unpaired as they continued searching for another resident with whom to pair. Wellness committee chairs suggested introducing peers to the unpaired residents. Unpaired residents were receptive to pairing with senior and junior colleagues. The quality improvement project was initiated in April 2020. The study subjects, including only the anaesthesiology residents in active training encompassing postgraduate years of training 1-4, were given an online survey 2-4 weeks later. This survey was designed to collect the participants’ experiences regarding the initiative and help direct the wellness committee toward the potential next steps of the subsequent PDSA cycle. 

Residents were encouraged to do daily check-ins for at least 2 weeks. The residents were surveyed to evaluate the frequency of check-ins, as well as to assess the impact of the implementation of the BUDDYS system on wellbeing. This included alleviating anxiety, reducing workload in terms of sharing information regarding COVID-19, helping the residents focus on family and personal time outside of work, or other aspects. The survey provided the option for free text comments if the resident wanted to provide additional feedback. Residents were given 2 weeks to complete the survey. As an essential part of communication among residents and the residency program, a list of resources was again provided to the residents in case concerns arose. Within the anaesthesiology training program, we implemented BUDDYS at no additional cost using few existing resources.

Because this study started as a quality improvement project, Institutional Review Board (IRB) approval was not required. Completing the survey was voluntary and anonymous, meaning consent was implied by participation in the survey; however, to proceed with sharing the results of the survey at the recommendation of our IRB, an IRB2 was approved as exempt (IRB #202001913). All residents were informed of the IRB and the publication of this quality improvement project. 

## Results

**Figure 1.**
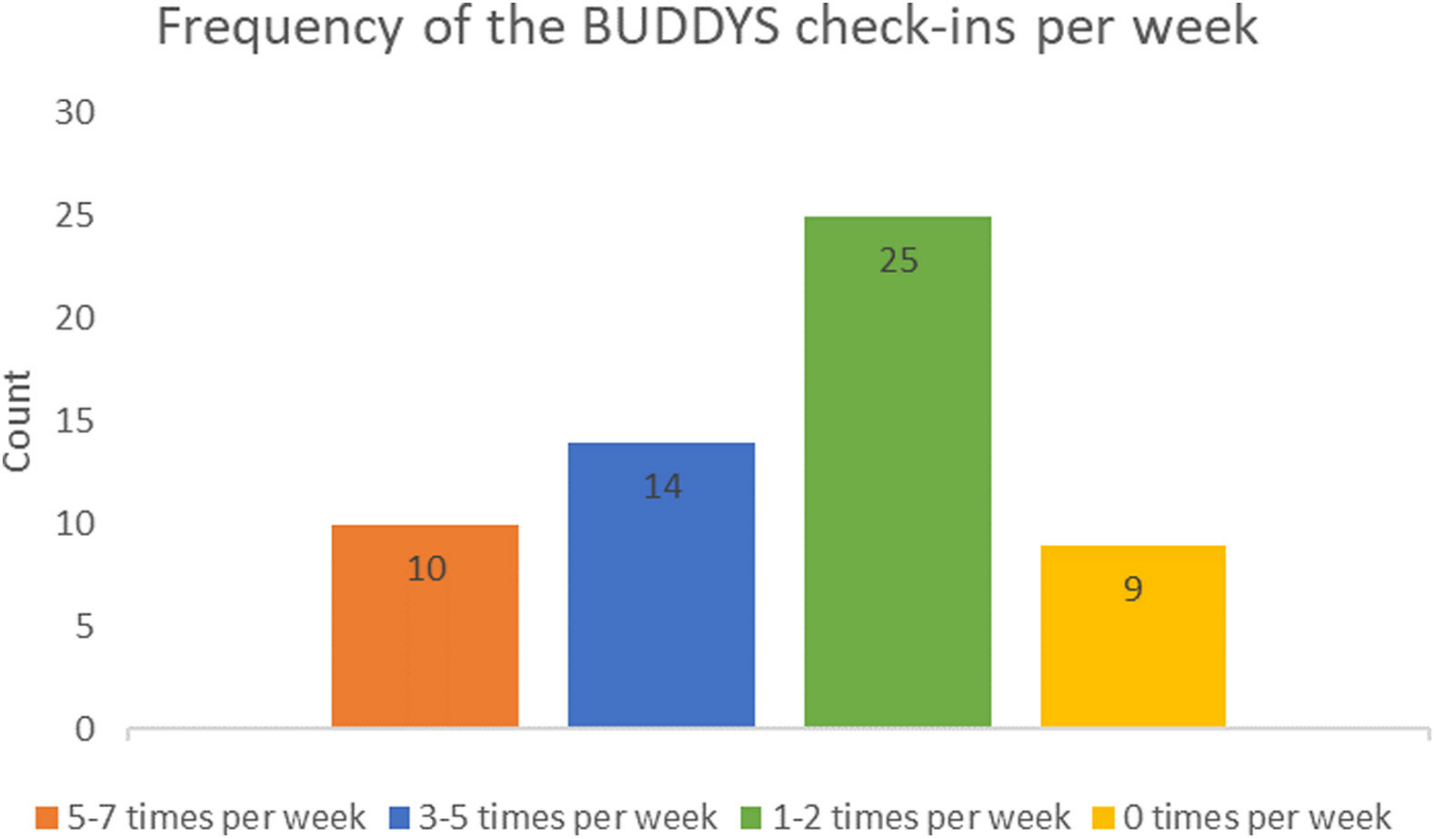
The number of times residents did weekly check-ins using the Building Dynamic Duos for Your Support (BUDDYS) system.

**Figure 2. f2-tjar-50-suppl1-s62:**
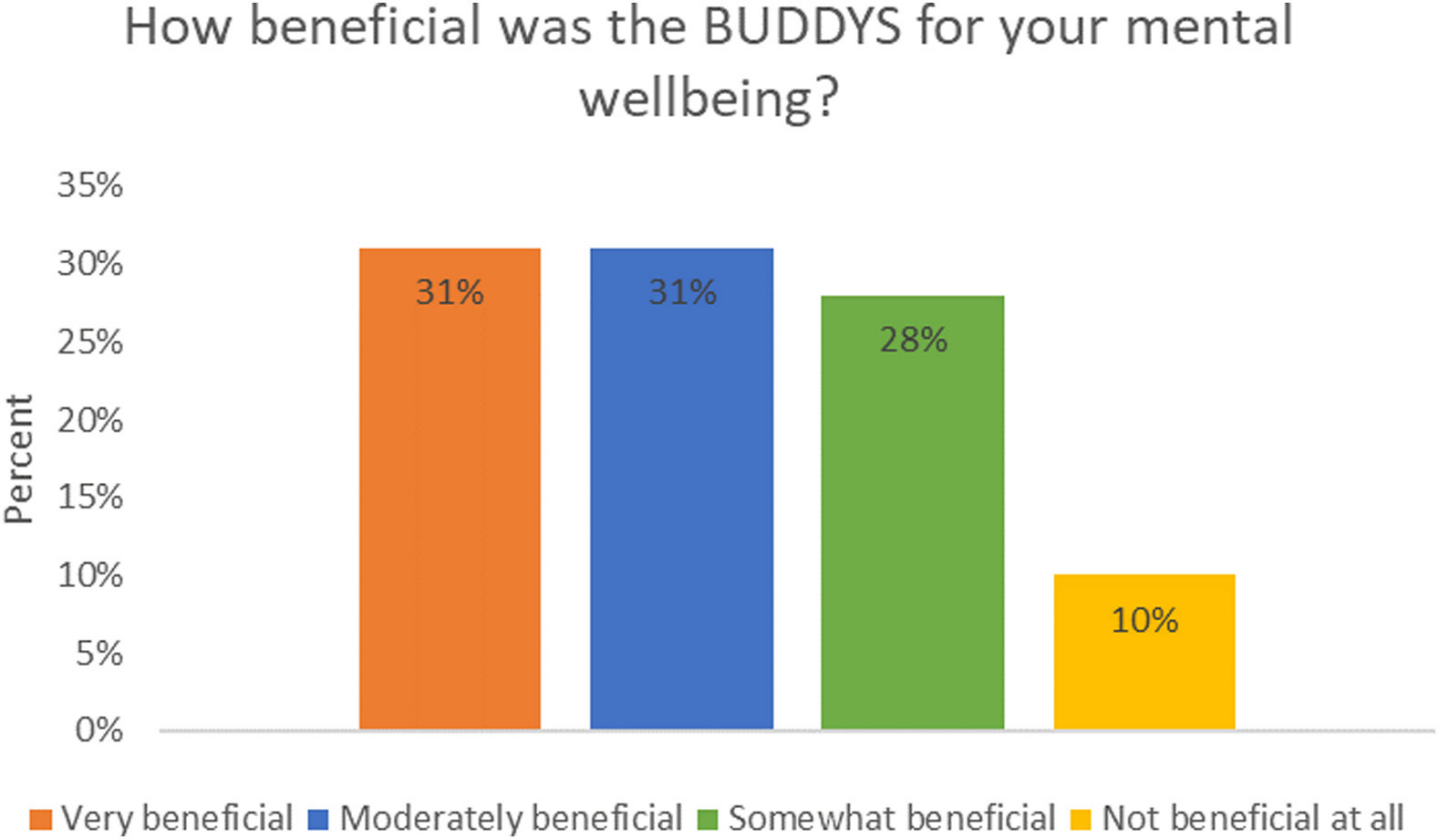
Resident responses to the survey question: “How beneficial was the BUDDYS for your mental wellbeing?” BUDDYS, Building Dynamic Duos for Your Support.

We surveyed 88 anaesthesiology residents 4 weeks after the introduction and 2 weeks following the completion of pairing within BUDDYS at our institution. Participation in the survey was voluntary and 58 (66%) residents participated. The survey focused on the establishment of the system in addition to its impact on residents’ overall wellbeing. The survey consisted of 4 questions (Figures 1-3). Most participants checked on their partners through BUDDYS weekly (Figure 1). The majority of the residents surveyed (60%) checked on their partner at least weekly, while 43% of the residents checked on their BUDDY 1-2 times per week, and 10 residents (17%) checked in almost daily (5-7 times per week). Adopting frequent check-ins as a habit was an essential step in implementing BUDDYS in preparation for the anticipated surge in the number of COVID-19 patients at our hospital. The overwhelming majority of those surveyed (90%) found the system helpful, responding that the buddy system was very beneficial, moderately beneficial, or somewhat beneficial to their overall mental wellbeing (Figure 2). Most of the participants (79%) responded that the BUDDYS system improved their overall mental wellbeing through alleviating anxiety, sharing the load of information related to COVID-19, and providing them with more time to spend with their families focusing on personal and family matters. 

Several of the free text comments in our survey supported the idea that these outcomes would likely result in better self-care and improved work-life balance. Participating residents responded positively in describing additional ways that BUDDYS helped with completing day-to-day self-care, benefiting from a support system, and promoting wellness. Their responses included comments such as “it is nice to talk to someone in the medical field about general non-medical topics” and “the system helped me get connected to someone in the program.” Residents also included ideas of how to incorporate BUDDYS for continued emotional, psychological, and mental health support for their buddy, for example by sending a letter of appreciation to the buddy. Although the majority of the comments were positive, there were 2 negative responses: “already had many friends in the program, did not need the buddy system” and “my buddy was a close friend that I would have been meeting with anyway and I was sharing personal information with anyway regardless of the BUDDYS system.” However, these comments reinforced the importance of a peer support system for a few of the residents who already used such a system. The implementation of BUDDYS across all classes established the idea of “peer support” and identified a peer to whom residents could reach out without the stigma of being labeled as “weak” when asking for help or when showing signs of mental or emotional distress.

## Discussion

Many clinicians are resistant to engage in wellness initiatives or address concerns related to burnout symptoms for fear of the stigma of seeking help or being recognized as non-resilient. During anaesthesiology residency, with the added uncertainty of the pandemic including unpredictable changes in workload, residents are frontline HCWs. They are at an increased risk of developing subclinical symptoms that can adversely affect their quality of life and impact their abilities to perform optimally. Subclinical symptoms include an ongoing sense of distress, worry, irritability, and disturbed sleep patterns. Many of these symptoms may result in interpersonal relationship difficulties that may be detrimental in an acute care work setting such as the operating room or the intensive care unit. A clear understanding of the physiological, cognitive, emotional, and interpersonal responses to working in a stressful environment remains key to balance resilience and burnout.^1^ Institutions have adopted different strategies to facilitate organizational and personal resilience and wellbeing. When facing psychological stressors, self-care and peer support are important coping strategies and both appear to be core interventions used in previous studies to promote wellbeing and resilience among HCWs.^1,7,9-13^

Peer support in the form of the buddy system has been introduced in graduate medical education in different reports that focused on the use of the buddy system to introduce medical students to the work environment during call hours for general surgery^14^ or as a method to introduce junior trainees to the work environment by pairing them with a senior resident as their buddy.^15^ In addition, the buddy system was introduced to HCWs as a method to work on patient safety initiatives^16^ or to improve the overall wellbeing of HCWs during non-crisis times.^17^ Peer mentor and buddy systems have also been reported to be effective in patient care and in enhancing patient compliance with treatments.^18^ In all of these instances, peer support has been shown to be beneficial, particularly to residents and students by helping them achieve their intended goals, whether those are training or introduction to a new or changing work environment. 

**Figure 4.**
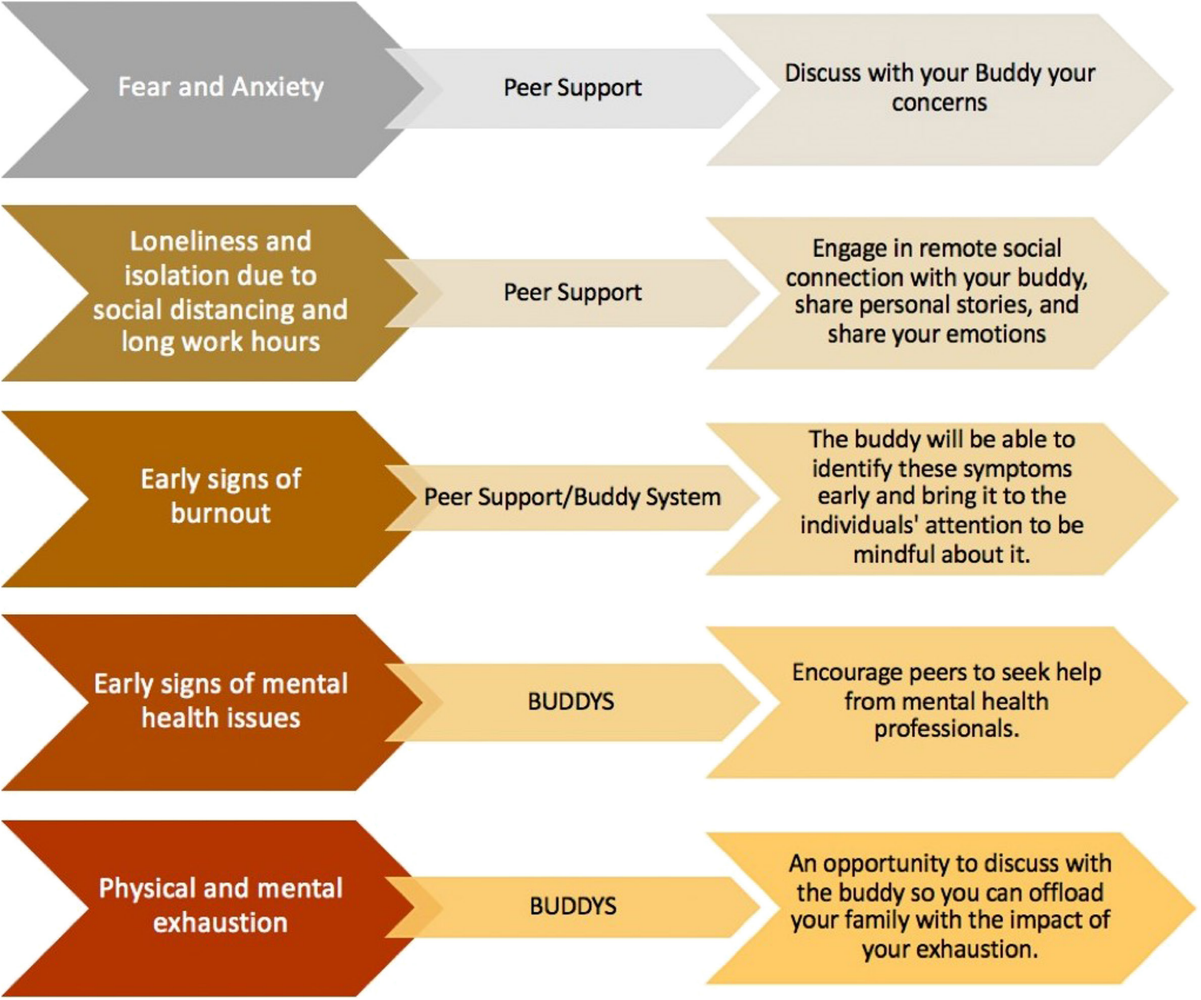
The use of the Building Dynamic Duos for Your Support (BUDDYS) system as a peer support intervention to promote personal resilience during the coronavirus disease 19 (COVID-19) pandemic.

The implementation of the BUDDYS system was a step to prepare residents for anticipated isolation, including residents who might have been quarantined due to exposures or documented infections, while ensuring that they continued to receive needed support (Figure 4). This implementation was also in response to the possible long work hours that might have been required during emergency situations such as the crisis of a pandemic. Such situations are not constrained by duty-hour rules from the Accreditation Council for Graduate Medical Education in the United States, which normally set a maximum of 80 hours per week. Caring for patients with COVID-19 or under investigation for COVID-19 created an additional level of distress as a result of the fear of infection to themselves and family members. The high level of anxiety imposed by the threat of an upcoming surge of cases and the potential impact on the unpredictability of the work environment also encouraged anaesthesiology program leadership and residents to be proactive in responding to the implementation of BUDDYS for peer support. The goals of BUDDYS at our residency program were for the partners to get to know each other, share COVID-19 and non-COVID-19 information on a daily and weekly basis, encourage daily check-ins regarding basic physiological needs and healthy habits (e.g., sleep, rest, exercise, and nutrition), help with mood checks, and share experiences and feelings.^2,19^

This regular contact allows residents to address and validate each other’s stressors on professional and personal levels. The main intention of this system was to encourage residents to find a partner with whom they could share stories, clinical experiences, and challenges that they faced during the COVID-19 pandemic because peers understand each other’s specific concerns through shared experiences. In addition, the system emphasized allowing work issues to remain at work, with the goal of preserving the home environment for relaxation and family time. 

The results of the survey showed that anaesthesiology trainees were open and enthusiastic about a new system to improve their overall wellbeing. Although one resident commented that they did not need BUDDYS and several residents commented that they already connected with their peers in a similar way, BUDDYS was a good opportunity for the majority of residents to connect with one another on a regular basis, which can be missed or overlooked in the middle of a pandemic and with the burden of social isolation. Many residents appeared introverted and reserved; the survey documented that the residents valued the emphasis on partnering with a colleague and providing and receiving emotional, cognitive, and mental support. All residents were introduced to the wellness and mental health resources that are available at our institution. They were also introduced to the next best steps to take in case a peer identified a colleague with concerning symptoms. 

The results of this quality improvement project highlight the potential implications of using a standardized approach for a peer support system among physician trainees in general and anaesthesiology residents in particular. The work environment for anaesthesiology residents usually involves high-acuity patient care in a fast-paced and stressful environment where providing resources that require daytime appointments may not be feasible. Instead, establishing a peer support program where residents can raise concerns, provide support, and observe one another for concerning signs of distress represents a feasible alternative; that is, talking to your buddy, who is often on a similar schedule, can be an easier first step than scheduling a call or an appointment with mental health professionals. A future longitudinal study involving the assessment of baseline mental health, burnout level, and work-life balance could be an appropriate next step for our residents. This future study will evaluate the impact of different wellness interventions and changes in the work environment on residents’ overall wellbeing.

All participants were working at the same institution, which may limit the generalizability of this group’s BUDDYS experience. However, many of the concerns our residents voiced would be relevant to all residents, particularly the need for support and recognition of limited channels to share experiences with others who work in the same environment. With the pandemic’s global impact, the BUDDYS system could have international relevance given that it was implemented without a financial cost and using available resources. Few participants in BUDDYS did not find it helpful; due to anonymity, the authors were unable to identify factors that might have led to this experience. In addition, since the survey was voluntary and anonymous, it might have failed to capture the introverted residents who were unwilling to formally participate in BUDDYS but participated and did not respond to the survey. Those introverted residents might be the ones who need peer support the most as it may be more difficult for them to reach out for help. Since this is our first quality improvement project establishing a wellness initiative, it was not feasible to assess the residents’ baseline behavioral conditions. 

Future studies may seek to identify those who derive the most benefits from BUDDYS and identify factors for the optimal generation of pairs. In addition, it is important to identify factors that led to the perception that BUDDYS did not have an overwhelmingly positive impact. This question involves self-awareness; it is possible that those who expressed that they did not derive benefit when surveyed may have experienced some beneficial effects. Further studies of whether the BUDDYS system impacted burnout, anxiety, or other psychological parameters with evaluation using specific tools and/or inventories (e.g., Maslach Burnout Inventory) remain an area for investigation. In addition, longitudinal studies could identify the longer-term impacts of this type of initiative.

## Conclusions

BUDDYS is a potential wellness tool for graduate medical education to introduce the concept of wellbeing, potentially prevent burnout, minimize anxiety, build resilience, and contribute to work-life balance goals for anaesthesiology residents. Despite being implemented during the COVID-19 pandemic, this system could benefit anaesthesiology residents even in non-crisis times. This system requires no additional financial commitment and the resources required should be readily available to residency training programs. BUDDYS benefited the majority of our residents in an anonymous survey. 

## Figures and Tables

**Figure 3.**
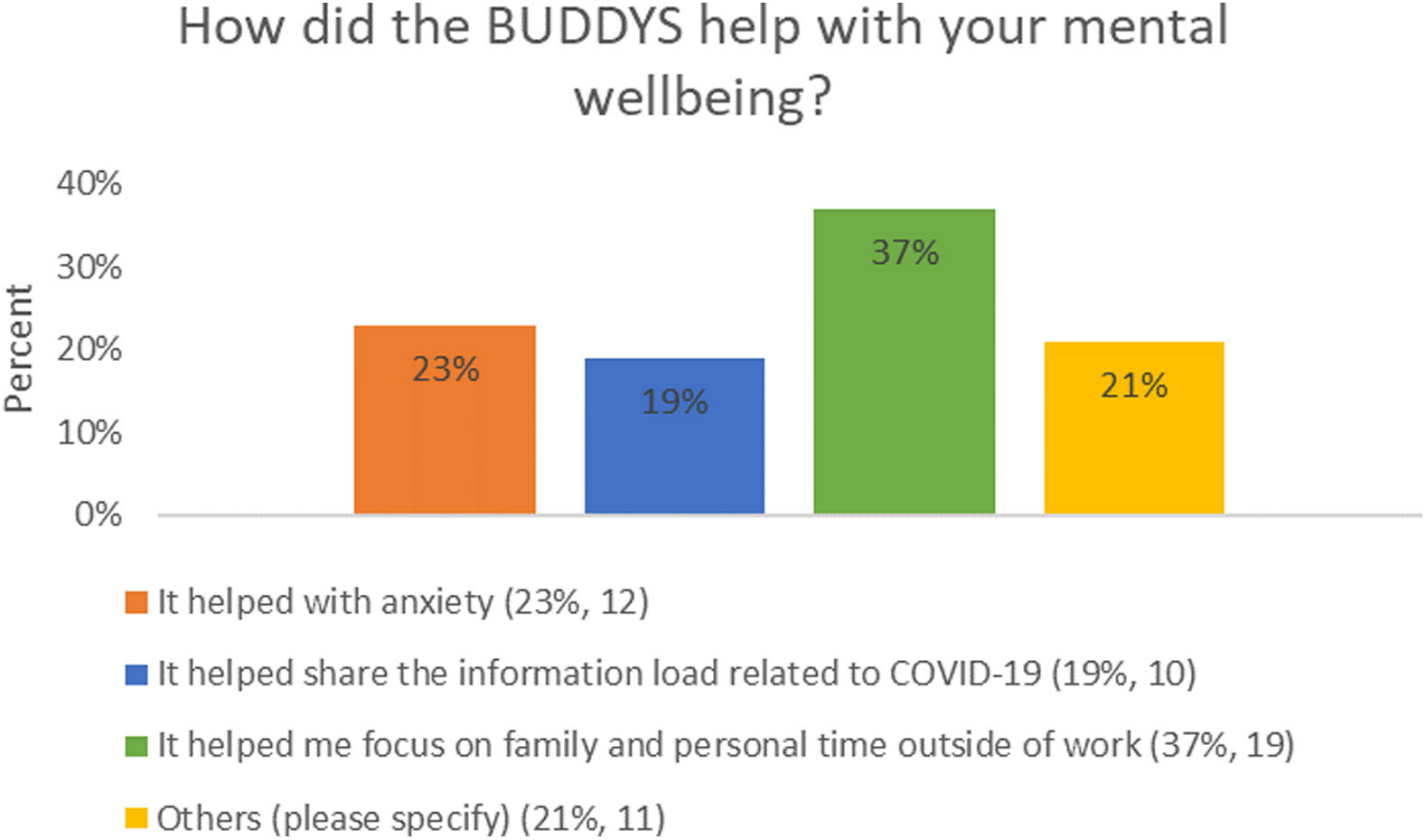
Resident responses to the survey question: “How did the BUDDYS help with your wellbeing?” BUDDYS, Building Dynamic Duos for Your Support.
